# A New View on an Old Debate: Type of Cue-Conflict Manipulation and Availability of Stars Can Explain the Discrepancies between Cue-Calibration Experiments with Migratory Songbirds

**DOI:** 10.3389/fnbeh.2016.00029

**Published:** 2016-02-23

**Authors:** Sissel Sjöberg, Rachel Muheim

**Affiliations:** Department of Biology, Lund UniversityLund, Sweden

**Keywords:** bird orientation, compass, calibration, migration, magnetic field, stars, sun, polarized light

## Abstract

Migratory birds use multiple compass systems for orientation, including a magnetic, star and sun/polarized light compass. To keep these compasses in register, birds have to regularly update them with respect to a common reference. However, cue-conflict studies have revealed contradictory results on the compass hierarchy, favoring either celestial or magnetic compass cues as the primary calibration reference. Both the geomagnetic field and polarized light cues present at sunrise and sunset have been shown to play a role in compass cue integration, and evidence suggests that polarized light cues at sunrise and sunset may provide the primary calibration reference for the other compass systems. We tested whether migratory garden warblers recalibrated their compasses when they were exposed to the natural celestial cues at sunset in a shifted magnetic field, which are conditions that have been shown to be necessary for the use of a compass reference based on polarized light cues. We released the birds on the same evening under a starry sky and followed them by radio tracking. We found no evidence of compass recalibration, even though the birds had a full view of polarized light cues near the horizon at sunset during the cue-conflict exposure. Based on a meta-analysis of the available literature, we propose an extended unifying theory on compass cue hierarchy used by migratory birds to calibrate the different compasses. According to this scheme, birds recalibrate their magnetic compass by sunrise/sunset polarized light cues, provided they have access to the vertically aligned band of maximum polarization near the horizon and a view of landmarks. Once the stars appear in the sky, the birds then recalibrate the star compass with respect of the recalibrated magnetic compass. If sunrise and sunset information can be viewed from the same location, the birds average the information to get a true geographic reference. If polarized light information is not available near the horizon at sunrise or sunset, the birds temporarily transfer the previously calibrated magnetic compass information to the available celestial compasses. We conclude that the type of cue-conflict manipulation and the availability of stars can explain the discrepancies between studies.

## Introduction

Migratory birds use multiple compass systems for orientation during migration, which include a magnetic compass (Wiltschko and Wiltschko, 1972, 1995), star compass (Emlen, 1970, 1975) and a sun/polarized skylight compass (Able, 1982; Moore, 1987; Schmidt-Koenig, 1990; Munro and Wiltschko, 1995). Because of changing relationships between the compass cues and altering cue availability due to weather conditions, time of day, season, and latitude, birds have to calibrate the different compasses with respect to a common reference on a regular basis (Bingman, 1983; Wiltschko et al., 1998; Able and Able, 1999; Muheim and Åkesson, 2002; Bingman et al., 2003; Muheim et al., 2006a).

Research on the integration and calibration of orientation cues has provided variable and often contradictory results, favoring either celestial or magnetic compass cues as the primary calibration reference (reviewed by Able, 1993; Åkesson, 1994; Wiltschko et al., 1997, 1998; Wiltschko and Wiltschko, 1999; Bingman et al., 2003; Muheim et al., 2003). Several studies had found a recalibration of the magnetic compass by sunset/polarized light cues (Bingman, 1983; Prinz and Wiltschko, 1992; Able and Able, 1995; Weindler and Liepa, 1999; Cochran et al., 2004). However, the majority of studies exposing birds to a cue conflict in funnels or cages that restricted the view of the horizon observed a dominance of the magnetic compass and a shift of the celestial “sunset” compass instead (see references in Muheim et al., 2006a). In a review on the literature, Muheim et al. (2006a) proposed that these contradictions between studies can be explained by differences in cue availability during the exposure to the cue conflict. Birds recalibrate their magnetic compass by polarized light cues, provided that they have access to a full view of the sky near the horizon at sunrise or sunset. In a series of cue-calibration experiments with migratory Savannah sparrows (*Passerculus sandwichensis*) and white-throated sparrows (*Zonotrichia albicollis*) they showed that the birds recalibrated their magnetic compass with respect to polarized light cues near the horizon at sunrise and sunset (Muheim et al., 2006b, 2007, 2009). As originally proposed by Phillips and Waldvogel for homing pigeons (Phillips and Waldvogel, 1988), birds use information from the vertically aligned e-vector of polarized light for the calibration of the magnetic compass. The sun compass has been suggested to be calibrated with respect to polarized light cues in a similar way (Moore and Phillips, 1988; Phillips and Moore, 1992). Averaging of sunrise and sunset calibration provides a true geographic reference, since the azimuth positions of the sun and polarized light at sunrise and sunset always are symmetrical to true geographic North (Phillips and Waldvogel, 1988; Muheim et al., 2006b, 2007; Muheim, 2011).

It has been suggested that differences in the magnetic field properties (e.g., variation in declination) between continents could explain the outcomes of different cue-calibration studies (Åkesson, 1994; Bingman et al., 2003; Liu and Chernetsov, 2012; Åkesson et al., 2015). The majority of studies finding recalibration of the magnetic compass had been carried out in North America, whereas many studies finding recalibration of the celestial compasses had been carried out in Europe or Australia. However, in studies carried out in North America the birds were usually provided a full view of the surroundings during the cue-conflict exposure, whereas the birds in Europe were exposed in orientation funnels, blocking the view of the horizon. To test whether the difference in outcome was due to the method (open cages vs. funnels) or location (North America vs. Europe or Australia), several recent studies investigated whether birds in Europe and Australia recalibrated their magnetic compass when they had a view of sunset cues near the horizon during the cue conflict. The majority of attempts found no recalibration of the magnetic compass, suggesting that a difference between location may be the more likely explanation for the discrepancy between studies (Wiltschko et al., 2008; Gaggini et al., 2010; Chernetsov et al., 2011; Schmaljohann et al., 2013; Åkesson et al., 2015). However, one recent study by Giunchi et al. (2014) found a recalibration of the magnetic compass after cue-conflict exposure in pied flycatchers (*Ficedula hypoleuca*) during spring migration in Italy. They combined orientation funnel experiments with radio tracking. The birds showed a clear recalibration of their magnetic compass when tested for magnetic compass orientation in orientation funnels after the cue-conflict exposure (Giunchi et al., 2014). However, the same individuals departed in an unchanged geographic direction when subsequently released and tracked by radio telemetry, suggesting no compass calibration. The authors explained these seemingly contradictory results between the different behavioral assays with the involvement of the star compass that remained unchanged during the cue conflict; their birds had access to the natural magnetic field and cues from the setting sun under a 90° shifted polarization pattern at sunset, but did not see any stars during the cue conflict (Giunchi et al., 2014). Once released, the birds simply followed their star compass calibrated prior to the cue conflict, and ignored the recalibrated magnetic compass.

Still, not all of the recent cue-calibration experiments named above can be solely explained by the involvement of a star compass. Thus, the question remains whether or not there is a unifying explanation for the outcomes of the different cue-calibration studies. In the present experiment, we used an automated radiotelemetry system at Falsterbo peninsula, Sweden, to test whether juvenile garden warblers (*Sylvia borin*) captured during autumn migration recalibrated their compasses when exposed to a horizontally shifted magnetic field with full view of the surroundings during sunset, but before the stars appeared.

## Methods

The study was performed at Falsterbo Bird Observatory, Falsterbo, Sweden (55°38′N, 12°82′) during the migratory seasons in autumn 2010 and 2012. Garden warblers are long-distance migrants with breeding ranges covering Scandinavia and northern Europe and wintering areas in tropical Africa (Cramp, 1992). They are expected to migrate in a southwesterly direction from southern Sweden according to ringing recoveries (Fransson and Karlsson-Hall, 2008).

All birds were caught during the mornings within the regular ringing scheme at Falsterbo Bird Observatory. Age was determined with the help of plumage characteristics. Only birds that had completed their post-juvenile molt were included in the study. Wing length was measured to the closest mm, fat score was visually estimated on a 0–9 scale (Pettersson and Hasselquist, 1985) and body mass was measured to the closest 0.1 g on a Pesola spring balance. The mass of the radio transmitter never exceeded the recommended upper weight limit of 5% of body mass. The experimental birds were kept indoors for 2–15 days in single bird cages from capture until the first night with favorable migration conditions (at least partly clear skies and light winds). The birds used as an extra control (not exposed to any experimental or control treatment; see below) were equipped with a radio transmitter and released within 1 h after capture.

The study was carried out in accordance with the recommendations of the Swedish Board of Agriculture. The protocol was approved by the Malmö-Lund ethical committee for scientific work with animals, Sweden (M 204-06 and M 27-10).

### Experimental treatment

We exposed and released 17 control birds (8 in 2010 and 9 in 2012) and 17 experimental birds (8 in 2010 and 9 in 2012). Cue-conflict exposures took place 13–29 Oct 2010 and 27 Aug–23 Sept 2012. Since the vast majority of birds caught at Falsterbo Bird Observatory arrive during the late night/early morning on the day of capture, it is difficult to know whether or not our birds had the opportunity to view the surroundings and update their compasses before capture. We used an 80 × 80 cm Helmholtz coil system powered by a 12 V car battery to shift the horizontal component of the magnetic field (for details see Sandberg et al., 1988). The experimental group was exposed to a deflected magnetic field (+90°) outdoors from 30 min before until 30 min after sunset, while the control group was exposed to the local magnetic field for the same period. During exposure, the birds were held in a wooden cage (48 × 33 × 32 cm) with fine-meshed plastic net on all four sides with four compartments made of the same net and covered on top. No more than one bird was held in each compartment, thus each bird had a clear view of the horizon in all directions. After the exposure, the birds were kept in a dark cage indoors for 1 h before a transmitter was glued (contact adhesive, Casco) to their back after cutting short a small area of feathers. We estimated fat score (see above) when the transmitter was attached. Immediately afterwards, the birds were released from the top of the lighthouse (25 m above ground) with at least 2 min between each release. The light of the lighthouse was switched off during the first 30 min after release to not bias the departure directions of the birds.

### Radio-telemetry setup and calculation of directions

We used an automatic radiotelemetry receiver system (SRX600) covering the peninsula and ID coded radio transmitters (NTQB-2, 0.35 g; all by Lotek Wireless, Newmarket ON, Canada). Directions were calculated as a circular mean weighing each signal by signal strength during a 10 min period. For each individual, we calculated the initial orientation from the 10 min period after release from the lighthouse receiver station (from where the birds were released), and the vanishing bearing, calculated as the signals of the last 10 min from the receiver station that the bird was last in contact with. For more details on setup, running regimes, and telemetry data analysis (see Sjöberg et al., 2015). For measurement bias and statistical uncertainty of the calculated vanishing bearings, see Sjöberg and Nilsson (2015).

In total, we included the initial orientation of 30 birds (15 controls and 15 experimentals) and the vanishing bearings of 23 birds (14 controls and 9 experimentals). Four birds (1 control and 3 experimentals) did not depart during the first night. Their vanishing bearings were not included in the analyses, because of the possibility that they could calibrate back their compasses during the following sunrise and/or sunset. However, their initial orientation was included in the analyses. Further, we removed the initial orientation of four birds (2 control birds and 2 experimental birds) and the vanishing bearings of 7 birds (2 control birds and 5 experimental birds) from the sample, because the birds were in contact with only one antenna during the 10 min periods, which does not allow calculating accurate directions. Exceptions were two vanishing bearings where the birds were in contact with one of the other receiver stations during their last 10 min, whereby the direction from that station was used instead. Of the 19 garden warblers released with transmitters without any exposure, only 13 departed during night time (between 18:00 and 06:00 CET) and are included in the analyses. For each group, we calculated the mean orientation using standard circular statistics (Batschelet, 1981). We used Watson U^2^-tests to test whether the orientation differed between experimental groups.

### Weather data

The Swedish Meteorological and Hydrological Institute (SMHI) collected data daily every third hour at the location of Falsterbo Bird Observatory. The weather reported at 19:00 CET was used as the weather during the exposure (sunset times varied between 17:50 and 19:14 CET during the study). Further, we used the weather reported closest to release to analyze the weather in relation to the initial orientation, and the weather closest to departure to analyze the weather in relation to the final vanishing bearings. Degree of overcast were visually estimated by human observers (0/8 = clear sky, 8/8 = total overcast; 9/8 = mist). Wind speed and direction were automatically measured.

### Meta-analysis of cue-calibration studies

We analyzed in detail all existing cue-conflict (exposure to cue conflict during orientation experiment) and cue-calibration (exposure to cue conflict before actual orientation experiment) studies published until 2015 to determine whether there were differences in magnetic field properties, access to celestial cues or any other factor that could explain the seemingly contradictory results between studies (for studies published before 2006, see Muheim et al., 2006a). We examined in more detail those studies in which pre-migratory or migratory birds were exposed to a cue conflict prior to orientation experiments under the following conditions: (1) the birds had a full view of the sky down to the horizon during the cue conflict, which is regarded as a prerequisite for magnetic compass calibration by polarized light cues (Muheim et al., 2006a,b), and (2) both the control and experimental groups were significantly oriented, and the orientation of the control group agreed with the seasonally expected migratory direction.

To investigate whether temporal or spatial variation in magnetic field properties at the study sites and along the migratory routes explained the different behaviors of the birds, we calculated the magnetic declination, inclination and total intensity, using the magnetic field model from the International Geomagnetic Reference Frame, IGRF2011. We restricted this analysis to one representative study per site and species (group) to avoid pseudo replication. We calculated the absolute differences in magnetic field properties between years from 1900 to 2015 to estimate the temporal variation of the magnetic field at each site. We determined the spatial variation of the magnetic field along a great circle route connecting the breeding and wintering areas for each species/site (IGRF 2011, year 2015). Thereby, we calculated the absolute changes in magnetic declination, inclination and total intensity for each step of 100 km along this migration route. Differences in mean variation were then compared between sites/species with different calibration strategies (magnetic vs. celestial compass as primary calibration source), migration strategies (short- to medium-distant migrant vs. long-distance migrant) and sites located on different continents (North America, Eurasia or Australia), using Kruskal-Wallis tests.

## Results

### Radio-tracking of experimental birds

**Figure 1:**
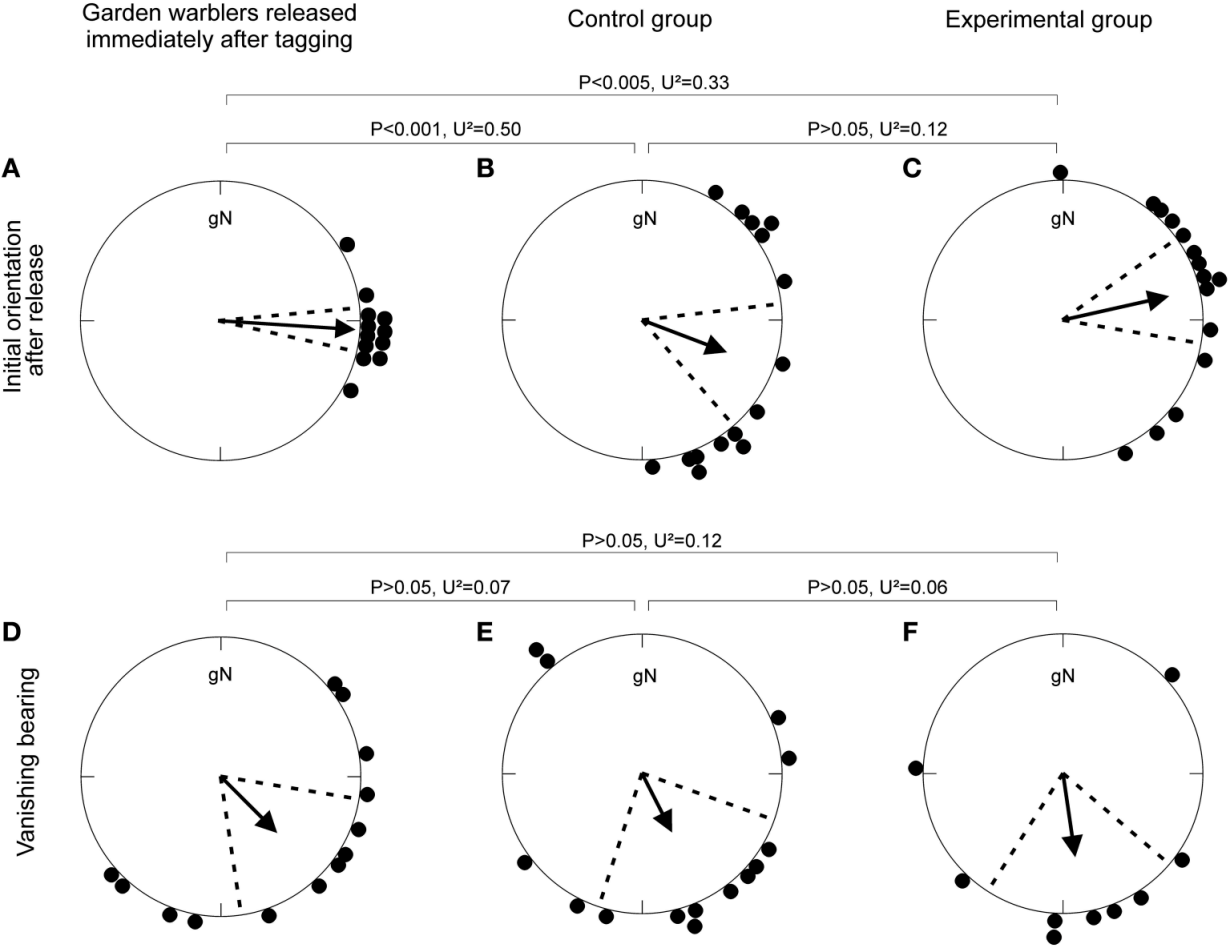
**Orientation of garden warblers followed by radio-tracking in Falsterbo, Sweden. (A–C)** Initial orientation of the first 10 min after release from the lighthouse receiver station for **(A)** birds released immediately after tagging during daytime, and **(B,C)** birds released at night after cue-conflict exposure under **(B)** the control condition and **(C)** the experimental condition. **(D–F)** Vanishing bearings of garden warblers **(D)** released immediately after tagging during daytime: **(E)** control and **(F)** experimental group.

The radio-tagged birds in both the control and experimental group initially departed from the lighthouse garden in easterly directions, and left Falsterbo peninsula in southeasterly directions (Figure 1, Table 1). Neither, the initial orientation immediately after release nor the final vanishing bearing differed between the control and the experimental groups. The 13 garden warblers released immediately after tagging differed in their initial response by being more concentrated than the control and experimental birds, but their mean vanishing bearing did not differ from the control or the experimental groups (Figure 1, Table 1).

**Table 1:** **Orientation of radio-tracked garden warblers at Falsterbo peninsula**.

**Treatment**		**Direction (°± 95% CI)**	***N***	***r***	**P (Rayleigh)**
Released immediately after tagging	Initial orientation	94.0 ± 9.0	12	0.97	<0.001
	Vanishing bearing	135.8 ± 36.2	13	0.57	0.011
Control group	Initial orientation	111.0 ± 28.0	15	0.65	<0.001
	Vanishing bearing	153.6 ± 44.4	14	0.48	0.038
Experimental group	Initial orientation	77.2 ± 22.6	15	0.78	<0.001
	Vanishing bearing	171.5 ± 40.9	9	0.61	0.032

All birds were released in winds <8 m/s and cloud coverage between 1/8 and 7/8. Even though the weather differed between release days, neither cloud cover, wind strength nor wind direction at release differed between the groups (Table S1). The weather was rather stable during the nights after release, and neither wind speed nor cloud coverage differed between the groups at departure (winds < 8 m/s, cloud coverage between 1/8 and 7/8; Table S1A). However, wind directions at departure differed between the groups, with more northwesterly winds when the control birds departed and more southerly winds when the experimental birds departed (Table S1B).

### Temporal and spatial variation of the magnetic field

**Figure 2:**
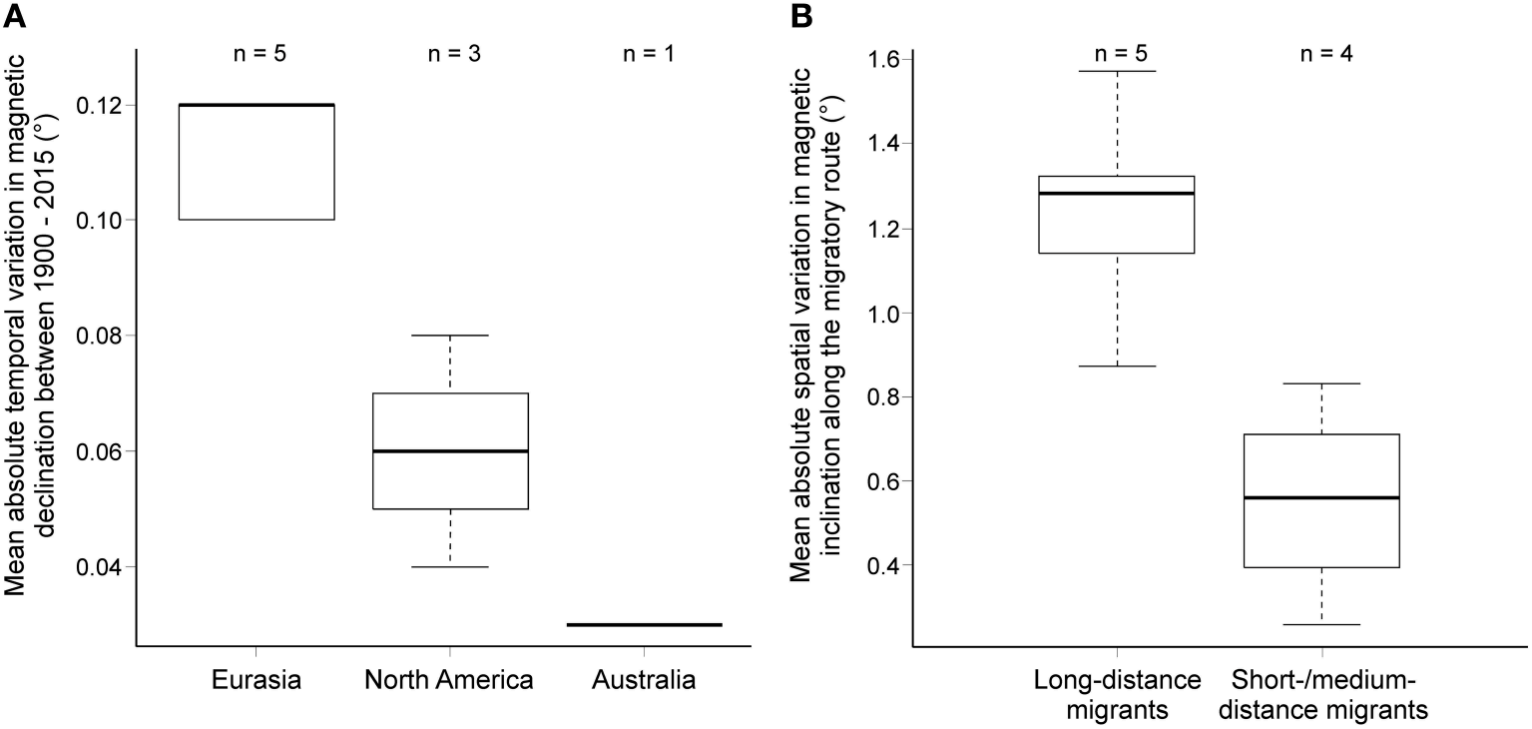
**(A)** Mean variation in magnetic declination between 1900 and 2015 for the different continents. **(B)** Mean variation in magnetic inclination by migration strategy (short- to medium-distance migrants vs. long-distance migrants). For details, see Table S2.

We found no difference in the absolute yearly variation in magnetic field properties (magnetic declination, inclination and total intensity) over the past 115 years between studies reporting calibration of the magnetic compass compared to studies finding recalibration of celestial compass(es) (Table S2A). However, the mean absolute yearly variation in declination varied significantly between continents (Figure 2A; Table S2A). In Europe, magnetic declination varied significantly more over the years than in North America and Australia, indicating that the magnetic field has been a much more unstable reference in the near past in Europe.

Spatial variation of the magnetic field along the migratory routes did not differ between groups with different calibration strategies or between continents. Magnetic inclination differed between migration strategies, being significantly larger in long-distance migrants than short- to medium-distance migrants (Figure 2B; Table S2B).

## Discussion

### Radio-tracking of experimental birds does not indicate recalibration of any compasses

The garden warblers in both the control and experimental group initially oriented toward easterly directions from the lighthouse during the first 10 min after release, but departed from Falsterbo peninsula in south-southeasterly directions, similar to the unmanipulated birds that were not exposed at sunset, but released immediately after tagging. The easterly initial orientation of the experimental group could indicate a recalibration of celestial cues relative to the magnetic field, but this is highly unlikely, since the control and the unmanipulated group showed a similar behavior. More likely, the birds initially exhibited a phototactic response toward the closest lights in the village and/or moved to the closest protection.

**Figure 3:**
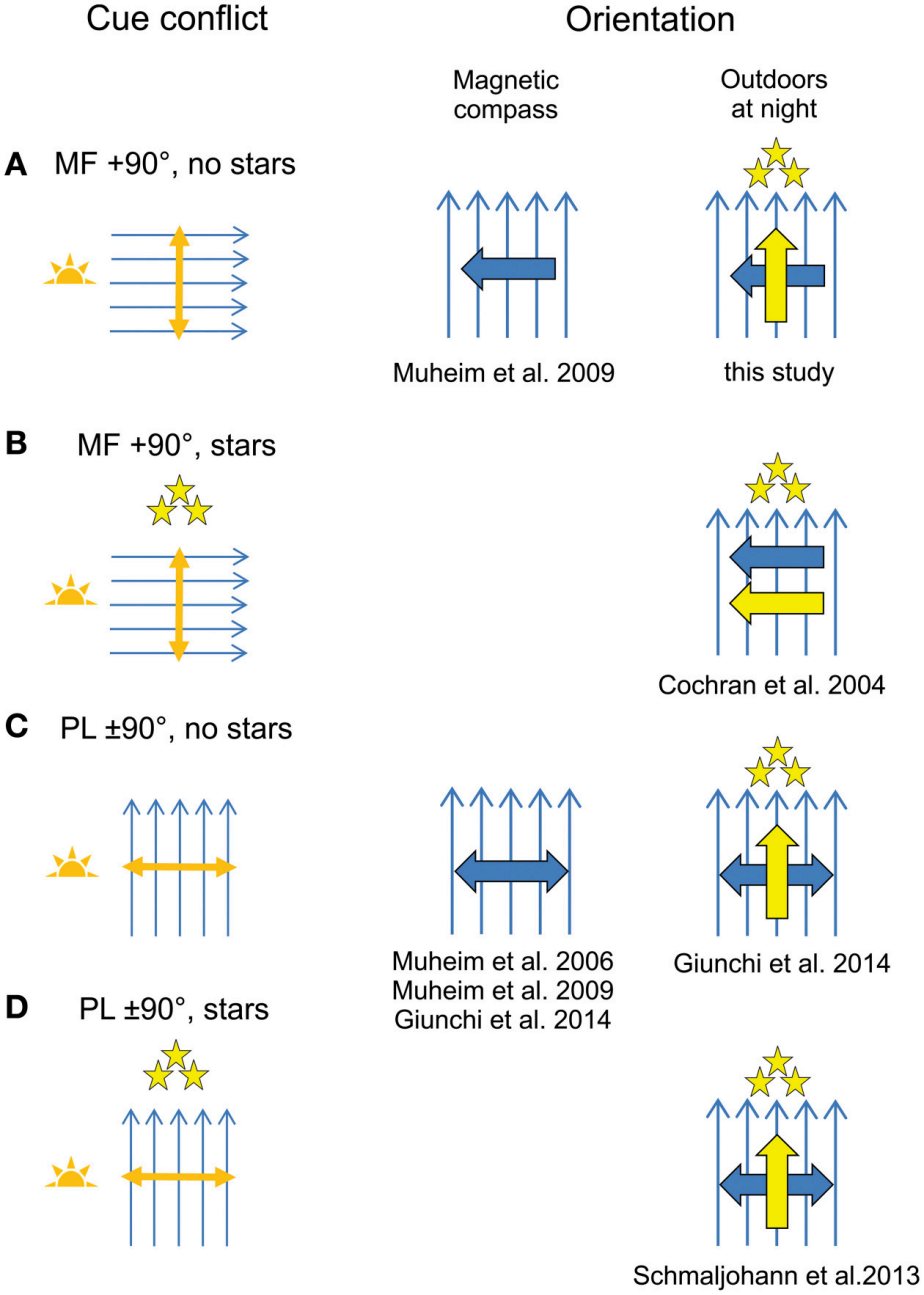
**Outcomes of recent cue-calibration experiments**. Included are studies that exposed migratory birds to a cue conflict between the magnetic field (MF; blue arrows) and polarized light (PL; orange double arrow) cues at sunrise or sunset with view of the horizon. **(A,B)** MF artificially shifted relative to the natural celestial cues (exemplified by a MF shifted 90° clockwise relative to its natural alignment). **(A)** MF shift in the presence of natural PL cues at sunset in the absence of stars; **(B)** MF shift in the presence of natural PL cues at sunset and stars. **(C,D)** ±90° shift of an artificial PL pattern relative to the natural MF at sunrise or sunset. **(C)**, Shift of PL pattern in the absence of stars; **(D)**, Shift of PL pattern in the presence of stars. Results of cue-conflict exposures as measured in orientation experiments in funnels (magnetic compass orientation; center column) or in release experiments outdoors at night (right column). The expected orientation is shown for birds tested for magnetic compass (large blue arrow or double arrow) and/or for birds released outdoors at night with access to the magnetic compass and star compass (large yellow arrow). Large double arrows are used when the direction of expected shift is axial (after exposure to a shift in the polarization axis). See Supplemental References and Table S3 for detailed methods on cue-conflict procedures used in the different studies.

We found no evidence for a compass recalibration in response to the exposure to a 90° shifted magnetic field for 1 h around sunset, even though the birds had a full view of the sky down to the horizon during the cue conflict (Figure 3A). These findings are consistent with recent orientation funnel and radiotelemetry studies carried out in Europe (Gaggini et al., 2010; Chernetsov et al., 2011; Schmaljohann et al., 2013; Giunchi et al., 2014; Åkesson et al., 2015), but are inconsistent with the studies carried out with North American passerines (Able and Able, 1993; Cochran et al., 2004; Muheim et al., 2006b, 2009). However, in view of the recent findings by Giunchi et al. (2014) we cannot exclude the possibility that the birds recalibrated their magnetic compass, but relied on their previously calibrated star compass to determine their departure direction. As was the case in the cue-conflict exposures carried out by Giunchi et al. (2014) (Figure 3C), our birds had no view of the starry sky during the cue conflict. They could have recalibrated their star compass after release, when the stars were visible, but by then there was no conflict between the magnetic reference, e.g., magnetic North, and the stellar reference, e.g., stellar North, anymore (see below).

### Temporal and spatial variation of magnetic field do not explain differences in calibration strategies between studies

The comparison of cue-calibration studies in which the birds were provided a full view of the horizon at sunrise or sunset did at first sight not reveal any systematic pattern that could explain the difference in the outcome of the different studies. The temporal variation of magnetic field properties over the past 115 years on the testing sites and the spatial variation along the migratory routes of the study species cannot explain why the birds recalibrated the magnetic compass in some and the celestial compass(es) in other studies. The birds did not appear to rely more heavily on the more stable of the compass(es), as has been previously argued (Åkesson, 1994; Bingman et al., 2003; Liu and Chernetsov, 2012; Åkesson et al., 2015). If that was true, the birds in Europe, where the temporal changes in magnetic declination were significantly larger than in North America (Figure 2A), should primarily rely on celestial cues and birds in North America on magnetic cues as the primary calibration source. However, the reverse is true, with birds studied in Europe relying more often on the magnetic than celestial compass(es) (χ^2^ test: χ^2^ = 7.76, *P* = 0.02, *df* = 3,1).

Similarly, we found no differences in the variation of magnetic field properties along the migration route of species using either one of the two calibration strategies. Instead, migration strategy explained some of the variation. Long-distance migrants, especially those species crossing the magnetic equator, experienced larger differences in magnetic inclination than short- to medium-distance migrants (Figure 2B). Long-distance migrants are overrepresented in Europe, but include species with both calibration strategies. Thus, the significant differences in the variation of magnetic field inclination are related to bird migration strategy and seem unrelated to calibration strategy. In summary, we found no relationship between calibration strategy and magnetic field properties, neither on a temporal or spatial scale, thus it is very unlikely that magnetic field properties explain why the birds at some sites recalibrate their magnetic compass, while birds at other sites recalibrate their celestial compass(es).

### A new view on an old debate

Is there a unifying explanation for the outcomes of the different cue-conflict and cue-calibration studies? A closer look at the exact conditions during the cue-conflict exposures reveals a consistent pattern that can explain all but a few studies (see below and Table S3). Based on the available evidence, we propose the compass hierarchy and calibration strategy outlined below, exemplified by a migratory bird stopping at a new stopover site after a night's flight.

Whenever a migratory bird lands at a new stopover site, encoding of both the magnetic and celestial compasses might be erroneous, since magnetic declination and the positions of sunrise and sunset will likely have changed since the last stopover site. To place the different compasses into register again, the bird will have to calibrate the different compasses with respect to a common reference, which we propose is the true geographic North, determined by averaging the vertically aligned e-vector of polarized light at sunrise and sunset.

#### Provisional recalibration of magnetic compass and subsequent recalibration of star compass

Since the bird can only determine true geographic North once it has seen both sunrise and sunset at the new stopover site, it will make a provisional recalibration of its magnetic compass during the first twilight period that it spends at the new site, with at least partially clear sky at either sunrise or sunset. It provisionally recalibrates its magnetic compass with respect to the vertically aligned band of maximum polarization near the horizon visible at either sunrise or sunset (whichever it sees first) at the new site. For a successful recalibration of the magnetic compass, a view of the vertically aligned e-vector of light at sunrise/sunset near the horizon is required. The sun compass has been suggested to be calibrated with respect to polarized light cues in a similar way (Moore and Phillips, 1988; Phillips and Moore, 1992). Magnetic compass orientation of birds that have carried out a provisional magnetic compass calibration is indistinguishable from the orientation of birds that have fully calibrated their magnetic compass (see below). Provisional calibrations of the magnetic compass can affect the bird's orientation at other times of day, e.g., a magnetic compass provisionally calibrated at sunrise has been shown to affect magnetic compass orientation at sunset (Muheim et al., 2006b, 2009).

As discussed by Muheim et al. (2006b, 2007) and Muheim (2011), the azimuth of the rising or setting sun at sunrise and sunset as it crosses the horizon depends highly on local topography. The band of maximum polarization on the other hand intersects the horizon vertically only at the exact times of sunrise and sunset, thus enables a much more accurate determination of the sunrise and sunset azimuths (Phillips and Waldvogel, 1988). However, as long as the bird has not seen both sunrise and sunset at the new stopover site, allowing it to average the information from the two times of day, it will not know the exact alignment of the geographic north-south axis. Therefore, we propose that it will use the stored deviation of the band of maximum polarization from the previous full recalibration for the provisional recalibration of the magnetic compass based on the polarized light information obtained at only one of the two times of day at the new stopover site. For example, if the bands of maximum polarization at the last stopover site where the bird observed both sunrise and sunset polarized light cues was aligned 38° counter clockwise and 38° clockwise of the geographic north-south axis, respectively, the bird will assume that this is also the case at this new location and provisionally recalibrate its magnetic compass according to this assumption. Thus, if weather conditions, for example, at the new stopover site only allow the bird to view the polarized light pattern at sunset, it will assume that the deviation of the geographic north-south axis is the same as the previous stopover site (i.e., 38° clockwise). This would allow it to update the magnetic compass, even though it has not yet been able to carry out a full recalibration with respect to the true geographic reference at the new site. The deviation of the band of maximum polarization at sunrise/sunset at the current stopover site will likely deviate from the 38° of the previous location, depending on the time passed and the difference in latitude between the current location and the last location where the bird saw both sunrise and sunset. This error can be corrected once weather conditions allow the bird to have access to the band of maximum polarization at both sunrise and sunset at the same stopover site (see below). However, since it is likely quite common that a bird will not be able to see both sunrise and sunset at some stopover sites, for example due to bad weather, it can still improve the calibration of the magnetic compass by using partial information at the current site, rather than continuing to rely on the earlier full recalibration.

**Figure 4:**
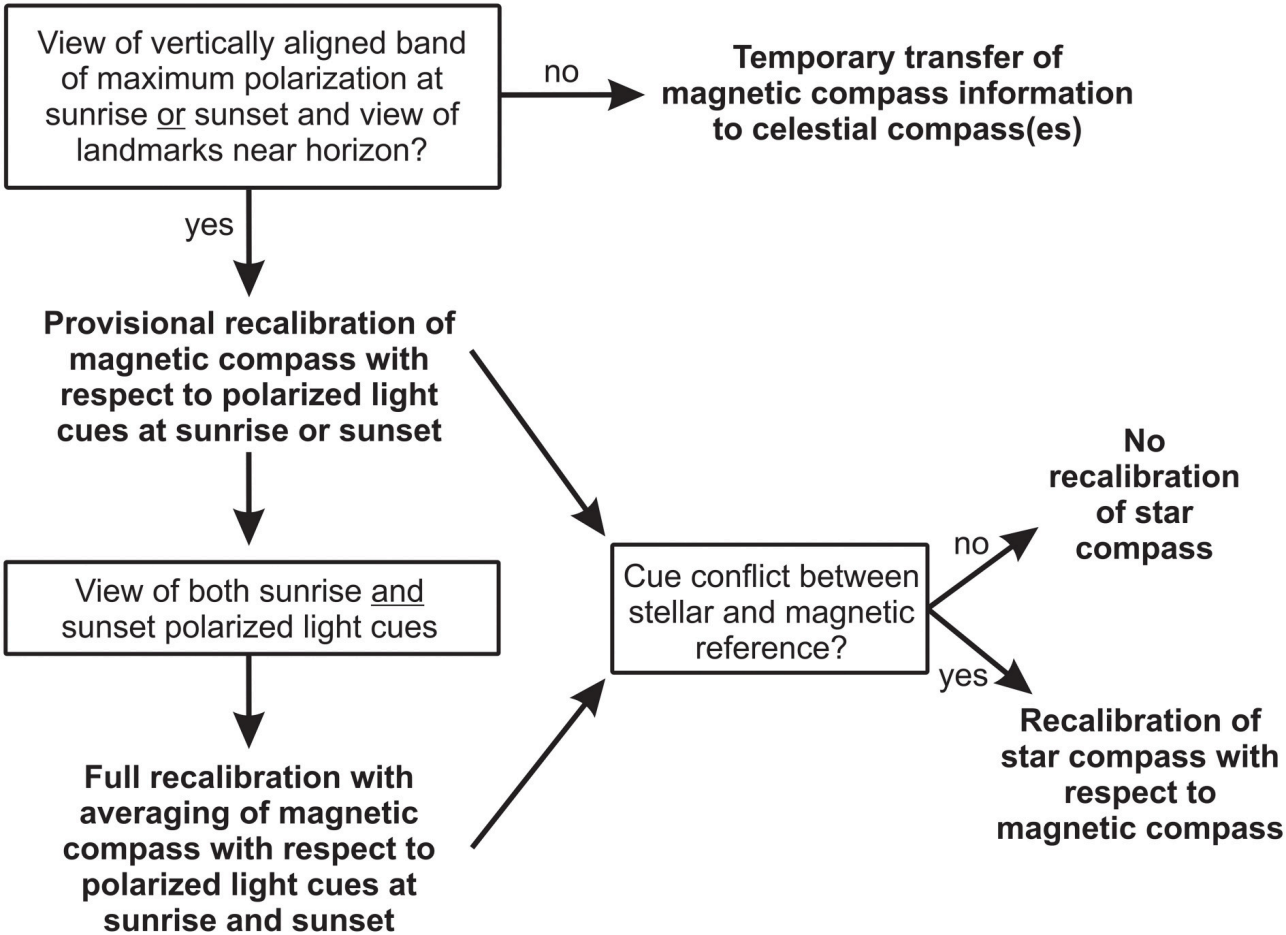
**Compass hierarchy and calibration strategy used by migratory birds during exposure to conflicting information between celestial and magnetic compass cues**. See main text for further explanation.

It is generally assumed that the star compass is recalibrated by the magnetic compass during migration (Emlen, 1967; Wiltschko and Wiltschko, 1975a,b, 1976; Beason, 1987; Bingman, 1987). Thus, when the stars in the sky become available, the bird calibrates its star compass with respect to the most recently recalibrated magnetic compass (Figure 4). We propose that the bird compares the deviation of the magnetic reference, e.g., magnetic North, with the stellar reference, e.g., stellar North, at the current stopover site with the value at the last visited site, similar to the comparison of the deviation of the band of maximum deviation from the true geographic reference between sites. If the difference between stellar and magnetic North has changed since the last calibration between the two compasses, the bird recalibrates its star compass with respect to the newly calibrated magnetic compass, irrespective of whether the magnetic compass was provisionally or fully recalibrated (see below). Star compass calibrations often occur with a delay of several hours or days of exposure to the cue conflict, indicating that the birds may not compare the two reference systems on a regular basis (Wiltschko and Wiltschko, 1975b, 1976; Beason, 1987).

#### Update of true geographic reference and full recalibration of magnetic and star compass

When the bird gets access to the complementary twilight period, so that it has seen both sunrise and sunset at the new site, it updates the geographic reference and uses this updated geographic reference to fully recalibrate the magnetic compass. To determine the true geographic reference at a new site and carry out a full recalibration of the magnetic compass, the bird needs to see the band of maximum polarization with access to landmarks at both sunrise and sunset (Figure 4). Since the azimuth positions of the sun and the band of maximum polarization at sunrise and sunset are symmetrical to the geographic north-south axis only at these two times of day, averaging of sunrise and sunset information provides a true geographic reference (Phillips and Waldvogel, 1988; Muheim et al., 2006b, 2007; Muheim, 2011). We suggest that the actual averaging of sunrise and sunset information is accomplished by transferring the alignment of the polarization axis to surrounding landmarks. During the first calibration period (sunrise or sunset), the bird transfers the alignment of the polarization axis to a distant landmark(s) and then averages this remembered alignment with the alignment of the polarization axis during the second calibration period viewed in the same surroundings (see below).

Once the true geographic reference is determined at the current site, the magnetic compass is recalibrated with respect to this true geographic reference, and the new deviation of the band of maximum polarization from the geographic north-south axis is stored in memory. Thus, for a full recalibration of the magnetic compass, the migratory bird needs access to the band of maximum polarization at both sunrise and sunset and a view of surrounding landmarks near the horizon. Full calibrations of the magnetic compass have been shown to be long lasting, if the bird remains on the same location or has no opportunity to update the calibration. Birds exposed for several weeks to a cue conflict outdoors before the start of the migration and tested during migration keep this calibration for an entire migration season (Bingman, 1983; Prinz and Wiltschko, 1992; Weindler and Liepa, 1999). However, full calibrations seem to be restricted to the same migration season, since autumn calibrations have been shown to not be transferable to spring (Able and Able, 1996).

### How does the proposed compass hierarchy and calibration strategy fit the available literature?

Birds experimentally exposed to cue conflicts between different compass cues often face unnatural conditions which not only include unnaturally large deviations between the orientation cues, but also restricted access to visual cues. We propose that a view of surrounding landmarks near the horizon, access to the vertically aligned band of maximum polarization at sunrise/sunset, and the availability of stars during the cue-conflict exposure each play an important role in the outcome of cue-conflict and cue-calibration studies (Figures 3, 4).

#### Importance of a view of surrounding landmarks near the horizon during cue conflict

A view of the surroundings seems crucial for successful provisional and full magnetic compass recalibrations. Birds exposed to a cue conflict during sunrise or sunset in funnels or cages that block the view of the horizon show no recalibration of the magnetic compass (for references, see Table 2 in Muheim et al., 2006a). Instead, they follow their previously calibrated magnetic compass and transfer the magnetic compass information to the available celestial cues (Figure 4). We call these responses transfers, since they seem to be temporary and specific to the time of day and cue-conflict situation. A transfer of magnetic compass information at sunset was not found to affect sun compass orientation the next morning (Wiltschko et al., 2001). Likewise, a transfer on one evening was not retained to the next evening (Sandberg et al., 1988; note, however, that this might be an extreme case, since the birds only saw about 90° of the sky around the zenith). Thus, directional information from the band of maximum polarization at sunrise or sunset, without access to landmarks near the horizon, appears not to be sufficient for a recalibration of the magnetic compass. The birds need access to visual cues, like landmarks, near the horizon to be able to successfully recalibrate their magnetic compass (provisional or full). The information from the band of maximum polarization at sunrise or sunset, however, can be used in transfers of magnetic compass information to celestial cues. We argue that these transfers do not occur naturally, but are the result of the unnatural conditions met by the birds in the orientation funnels that block access to visual landmark cues near the horizon. If deprived of landmarks, the birds instead follow the magnetic compass that they recalibrated at the last stopover site, and temporarily transfer this information to the available celestial cues. Presumably, it is more adaptive for a bird to temporarily transfer the magnetic compass information calibrated at the previous stopover site to the available celestial cues, when a recalibration of the magnetic compass is not possible, than not to recalibrate any of the compasses, since otherwise the bird will be left with two (or more) compasses pointing in different directions.

#### Importance of access to the vertically aligned band of maximum polarization at sunrise/sunset during cue conflict

As outlined above, a view of the vertically aligned band of maximum polarization at sunrise/sunset near the horizon is required for a successful (provisional or full) recalibration of the magnetic compass. This explains why birds exposed to a cue conflict between magnetic and celestial compass cues at times of day that do not include sunrise or sunset (e.g., during the night or at midday) did not recalibrate any of their compasses, despite of a full view of the horizon (Able and Able, 1990; Muheim et al., 2006b, 2007). It is interesting to note that we don't see any temporary transfers of magnetic to celestial compass information under these conditions, suggesting that celestial cues other than the vertically aligned band of maximum polarization at sunrise or sunset cannot be used for calibrations or transfers of compass cues.

One cue-conflict study appears to contradict the importance of the band of maximum polarization at sunrise and sunset in the transfer of compass cues. Åkesson et al. (2002) found a transfer of magnetic to celestial compass cues after they had exposed birds to a 90°-shifted magnetic field with full view of the surroundings during the early afternoon. However, the birds had previously been exposed to the same type of cue conflict in funnels at sunset. Thus, it is more likely that they transferred the magnetic compass information to the celestial compass(es) during the sunset cue-conflict exposure, since the findings of other studies suggest that they would not have been able to make any transfers or calibrations during the subsequent cue-calibration exposure in the afternoon.

#### Presence and availability of stars during the cue conflict

As outlined above, birds that have averaged the polarization axis near the horizon at sunrise and sunset, and used this updated geographic reference to recalibrate the magnetic compass, will recalibrate the star compass with respect to this new magnetic compass reference. However, such star compass recalibrations only take place, if stars are available for calibration during the cue-conflict exposure and if the star compass and magnetic compass reference are in conflict. This can explain the different outcomes of recent cue-calibration experiments, as suggested by Giunchi et al. (2014). Cochran et al. (2004) exposed birds to a 90°-shifted magnetic field with full view of the surroundings at sunset until the stars appeared in the sky (Figure 3B). As a result, the birds recalibrated their star compass with respect to the changed relationship between stellar and magnetic North. In the current study, the birds were exposed to the same type of cue conflict as in Cochran et al. (2004), but they never saw the stars during the exposure. Thus, our birds never experienced a conflict between stellar and magnetic North. Once released, they followed their previously calibrated star compass and thus showed no response to the cue-conflict exposure (Figure 3A). They could have recalibrated their star compass after release, when the stars were visible, but by then there was no longer a conflict between stellar and magnetic North. Since we did not specifically test our birds for magnetic compass orientation, we can only assume that they recalibrated their magnetic compass as response to the cue-conflict exposure, as has been shown by Giunchi et al. (2014). That birds compare the stellar and magnetic references, as opposed to the star and magnetic compass courses, also explains why the Northern wheatears (*Oenanthe oenanthe*) tracked by radiotelemetry after a cue-conflict exposure showed no sign of recalibration (Figure 3D; Schmaljohann et al., 2013). These birds had access to star information during the cue conflict, but since the birds were exposed to a shifted polarized light pattern relative to the natural sky and natural magnetic field, the relationship between the magnetic field and the stars remained unchanged.

#### Exceptions to modified theory on compass cue hierarchy

Two notable exceptions to the proposed compass hierarchy and calibration strategy are the studies by Wiltschko et al. (2008) and Chernetsov et al. (2011). In both studies, the birds were exposed to a 90°-shifted magnetic field with full view of the vertically aligned band of maximum polarization near the horizon at sunrise/sunset, including surrounding landmarks, until the appearance of stars. When subsequently tested for magnetic compass orientation (Wiltschko et al., 2008) or released and followed by radiotelemetry (Chernetsov et al., 2011), however, the birds appeared not to have recalibrated any of their compasses. According to our theory, the birds in both studies should have been able to recalibrate their magnetic compass from the sunrise/sunset polarized light cues and subsequently the star compass from the newly recalibrated magnetic compass. In the study by Chernetsov et al. (2011), there is the possibility of a topographic bias in the departure directions of the song thrushes (*Turdus philomelos*) released on/near the Courish spit, Russia, as indicated by the unusually well-concentrated orientation of the radio-tracked birds (Chernetsov et al., 2011). More likely, however, as argued earlier (Muheim et al., 2008), birds might not pay attention to changed cues after prolonged exposure to the natural cues in the same area. In both Wiltschko et al. (2008) and Chernetsov et al. (2011), the birds were kept in outdoor aviaries with full view of the natural celestial cues prior to the cue-conflict exposures in the same area. If, as suggested here, the recalibration of the magnetic compass and the averaging of sunrise and sunset information is accomplished by transferring the alignment of the polarization axis to surrounding landmarks, the birds might not have paid attention to calibration cues once a full calibration had been transferred to local landmarks, as long as the same landmarks were available. This suggests a common explanation for the failure of birds to recalibrate their magnetic compass when exposed to a cue conflict in familiar surroundings or with polarized light cues obscured from the region of the sky near the horizon.

Two additional studies do not follow the proposed compass hierarchy and calibration strategy. Able and Able (1993) found recalibration of the magnetic compass in Savannah sparrows, even though the birds were exposed to the cue conflict in orientation funnels, and therefore had no view of the polarized light cues near the horizon. In another experiment (Able and Able, 1990; Table S3), the birds did not recalibrate their magnetic compass despite a full view of polarized light cues near the horizon at sunrise and sunset. We can only speculate about why the results of these two studies appear contradictory. In view of the otherwise consistent responses found in Savannah sparrows, we believe they are outliers. Two factors that might help to explain these apparent exceptions could be (1) sources of radio-frequency interference (e.g., near antennas, elevator motors, etc.) on top of the building where some of Able's exposures were carried out could have altered or eliminated the birds' perception of the magnetic field (Engels et al., 2014), or (2) exposure in Emlen funnels at locations where visual landmarks were visible above the edge of the funnels.

## Conclusions and outlook

We have proposed a modified theory of the compass hierarchy and calibration strategy used by migratory birds, and likely also non-migratory species (see Phillips and Waldvogel, 1988; Waldvogel et al., 1988), to calibrate the different compasses. The majority of published cue-calibration studies can be explained by this revised theory, but new studies are necessary to test the predictions outlined here. The proposed importance of landmarks for the recalibration of the magnetic compass and averaging of sunrise/sunset polarized light cues can be tested by exposing birds to a cue conflict at sunrise or sunset in funnels or cages covering all surrounding natural landmarks, and providing one group of birds an artificial landmark in the same type of surroundings. The group of birds provided with landmarks would then be expected to recalibrate its magnetic compass, while the group of birds deprived of all landmarks would then be expected to transfer the previously calibrated magnetic compass information to celestial cues. As a consequence, the two groups of birds should shift their orientation in opposite directions. The proposed importance of stars in the outcome of cue-conflict experiments can be tested by exposing one group of birds to a cue conflict in a shifted magnetic field for 1 h at sunset, and another group for 1 h from sunset to the appearance of stars. Once released and/or tested in a vertical magnetic field with access to the starry sky, the first group should follow the unchanged star compass, while the second group should follow the newly recalibrated star compass.

It will be important, however, to avoid some of the pitfalls present in earlier studies, e.g., allowing birds to access the natural relationship between orientation cues and surrounding landmarks prior to the cue-conflict exposures (cf. Wiltschko et al., 2008; Chernetsov et al., 2011), not giving them enough time for calibration at sunrise/sunset (Åkesson et al., 2015) or giving them calibration information that is conflicting between sunrise and sunset (Gaggini et al., 2010). Also, the use of release experiments to study compass hierarchies poses significant problems in the interpretation of results, since it is impossible to know which compass(es) the birds are using when they depart (see also Wiltschko et al., 2008). Cochran et al. (2004), for example, assumed that their birds used the magnetic compass when released from the exposure cages, but it is equally likely that they used the star compass. Combining funnel experiments with release experiments as done in Giunchi et al. (2014) provides much more valuable information, since magnetic compass and celestial compass orientation can be tested separately.

## Author contributions

SS and RM initiated the study and planned the experiments, SS collected the data and analyzed the data with input from RM, RM developed the theory with input from SS, SS and RM wrote the manuscript.

## Funding

This work was financially supported by grants from the Swedish Research Council (2007-5700 and 2011-4765 to RM), from the Royal Physiographical Society in Lund, The Crafoord Society (2011-0945), and from Lunds Djurskyddsfond. The project has also received financial support from a Linnaeus grant (no. 349-2007-8690) to the Centre of Animal Movement Research from the Swedish Research Council and Lund University.

### Conflict of interest statement

The authors declare that the research was conducted in the absence of any commercial or financial relationships that could be construed as a potential conflict of interest.
