# Supplementary material for: A New View on an Old Debate: Type of Cue-Conflict Manipulation and Availability of Stars Can Explain the Discrepancies between Cue-Calibration Experiments with Migratory Songbirds
Source: Front Behav Neurosci. 2016 Feb 23;10:29. doi: 10.3389/fnbeh.2016.00029 (PMC4763052; doi:10.3389/fnbeh.2016.00029)
Supplement: Supplementary file 1 [file Table1.DOCX]

**Supplementary table**

# Table S1A. Weather conditions at Falsterbo peninsula

| *Weather variable* | *Treatment* | *Mean* ± STD | *F* | *P (ANOVA)* | *df* |
| --- | --- | --- | --- | --- | --- |
| Wind speed at release | Control group | 5 ± 1.5 m/s |  |  |  |
|  | Experimental group | 5 ± 2.3 m/s | 0 | 1 | 1 |
| Cloud cover at release | Control group | 3.1/8 ± 1.9/8 |  |  |  |
|  | Experimental group | 3.8/8 ± 2.1/8 | 1.03 | 0.32 | 1 |
| Wind speed at departure | Control group | 4.7 ± 1.3 m/s |  |  |  |
|  | Experimental group | 4.7 ± 2.9 m/s | 0.005 | 0.94 | 1 |
| Cloud cover at departure | Control group | 3.6/8 ± 2.8/8 |  |  |  |
|  | Experimental group | 4.1/8 ± 2.0/8 | 0.19 | 0.67 | 1 |

# Table S1B. Weather conditions at Falsterbo peninsula

| *Weather variable* | *Treatment* | *Mean* ± STD (°) | *r* | *P (Rayleigh)* | *N* | *U^2^* | *P* | *df* |
| --- | --- | --- | --- | --- | --- | --- | --- | --- |
| Wind directions at release | Control group | 269.9 ± 136.0 | 0.06 | 0.95 | 15 |  |  |  |
|  | Experimental group | 195.3 ± 86.1 | 0.32 | 0.21 | 15 | 0.17 | >0.5 | 15, 15 |
| Wind directions at departure | Control group | 310.3 ± 122.5 | 0.10 | 0.87 | 14 |  |  |  |
|  | Experimental group | 172.4 ± 73.2 | 0.44 | 0.17 | 9 | 0.19 | <0.5 | 9,14 |
