# Supplementary material for: A New View on an Old Debate: Type of Cue-Conflict Manipulation and Availability of Stars Can Explain the Discrepancies between Cue-Calibration Experiments with Migratory Songbirds
Source: Front Behav Neurosci. 2016 Feb 23;10:29. doi: 10.3389/fnbeh.2016.00029 (PMC4763052; doi:10.3389/fnbeh.2016.00029)
Supplement: Supplementary file 2 [file Table2.DOCX]

**Supplementary table**

# Table S2A. Temporal variation in magnetic field properties

| *Species* | *Place* | *Continent* | *Latitude (°)* | *Longitude (°)* | *Migratory strategy (distance)* | *Calibration strategy (primary compass reference)* |  | *Absolute variation between years from 1900 to 2015 (mean ± STD)* | | |
| --- | --- | --- | --- | --- | --- | --- | --- | --- | --- | --- |
|  |  |  |  |  |  |  |  | *Decl. (°)* | *Incl. (°)* | *F (nT)* |
| Song thrush | Russia | Eurasia | 55.1 | 20.8 | short/medium | magnetic |  | 0.10 ± 0.04 | 0.02 ± 0.02 | 27.0 ± 10.4 |
| Garden warbler | Sweden | Eurasia | 55.4 | 12.8 | long | magnetic |  | 0.12 ± 0.04 | 0.02 ± 0.01 | 25.1 ± 9.5 |
| Northern wheatear | Helgoland | Eurasia | 54.1 | 7.9 | long | magnetic |  | 0.12 ± 0.04 | 0.01 ± 0.01 | 24.0 ± 9.0 |
| Pied flycatcher | Latvia/Italy | Eurasia | 56.9 | 23.1 | long | celestial |  | 0.10 ± 0.04 | 0.02 ± 0.02 | 26.9 ± 10.6 |
| Pied flycatcher | Germany | Eurasia | 50.1 | 8.7 | long | celestial |  | 0.12 ± 0.04 | 0.01 ± 0.01 | 24.3 ± 9.2 |
| Sparrow sps. | New York | North America | 46.6 | -73.8 | short/medium | celestial |  | 0.04 ± 0.03 | 0.05 ± 0.04 | 66.8 ± 40.4 |
| Savannah sparrow | Alaska | North America | 60.8 | -161.8 | short/medium | celestial |  | 0.08 ± 0.08 | 0.02 ± 0.01 | 23.4 ± 18.8 |
| *Catharus* thrushes | Illinois | North America | 40.1 | -88.2 | long | celestial |  | 0.06 ± 0.05 | 0.04 ± 0.02 | 70.6 ± 36.5 |
| Australian silvereye | Australia | Australia | -30.5 | 151.6 | short/medium | magnetic |  | 0.03 ± 0.02 | 0.02 ± 0.01 | 15.2 ± 12.4 |
|  | |  | |  |  | |  |  |  |  |
| *P-values (Kruskal-Wallis) by* | |  | |  | Calibration strategy | |  | 0.53 | 0.35 | 0.33 |
|  |  |  | |  | Continent | |  | **0.04** | 0.12 | 0.24 |
|  |  |  | |  | Migratory strategy | |  | 0.06 | 0.28 | 0.62 |

Decl. = declination; Incl. = inclination; F = Total intensity of magnetic field

# Table S2B. Spatial variation in magnetic field properties

| *Species* | *Place* | *Continent* | *Migratory strategy (distance)* | *Calibration strategy (primary compass reference)* | *Latitude at breeding site (°)* | *Longitude at breeding site (°)* | *Latitude at wintering site (°)* | *Longitude at wintering site (°)* | |  | *Absolute variation between 100 km steps along the migratory great circle route (mean ± STD)* | | |
| --- | --- | --- | --- | --- | --- | --- | --- | --- | --- | --- | --- | --- | --- |
|  |  |  |  |  |  |  |  |  |  |  | *Decl. (°)* | *Incl. (°)* | *F (nT)* |
| Song thrush | Russia | Eurasia | short/medium | magnetic | 63 | 35 | 45 | 5 | |  | 0.45 ± 0.22 | 0.53 ± 0.13 | 237.8 ± 19.2 |
| Garden warbler | Sweden | Eurasia | long | magnetic | 65 | 20 | 9 | 0 | |  | 0.16 ± 0.12 | 1.28 ± 0.63 | 307.9 ± 75.8 |
| Northern wheatear ^1)^ | Helgoland | Eurasia | long | magnetic | 65 | -20 | 54 | 8 | |  |  |  |  |
|  |  |  |  |  | 54 | 8 | 10 | -5 | |  | 0.31 ± 0.35 | 1.13 ± 0.67 | 283.5 ± 105.7 |
| Pied flycatcher | Latvia/Italy | Eurasia | long | celestial | 62 | 25 | 9 | 8 | |  | 0.16 ± 0.10 | 1.33 ± 0.63 | 309.8 ± 71.0 |
| Pied flycatcher | Germany | Eurasia | long | celestial | 52 | 9 | 7 | 1 | |  | 0.10 ± 0.05 | 1.57 ± 0.56 | 327.2 ± 71.9 |
| Sparrow sps. | New York | North America | short/medium | celestial | 50 | -73 | 30 | -83 | |  | 0.49 ± 0.09 | 0.59 ± 0.08 | 352.0 ± 100.8 |
| Savannah sparrow | Alaska | North America | short/medium | celestial | 62 | -162 | 40 | -120 | |  | 0.28 ± 0.18 | 0.26 ± 0.11 | 125.7 ± 87.1 |
| *Catharus* thrushes | Illinois | North America | long | celestial | 50 | -95 | 3 | -75 | |  | 0.13 ± 0.12 | 0.87 ± 0.24 | 488.3 ± 93.6 |
| Australian silvereye | Australia | Australia | short/medium | magnetic | -42 | 146 | -28 | 153 | |  | 0.16 ± 0.01 | 0.83 ± 0.07 | 493.5 ± 39.5 |
|  | |  | |  |  |  | | |  |  |  |  |  |
| *P-values (Kruskal-Wallis) by* | |  | | Calibration strategy | |  | | |  |  | 0.45 | 0.81 | 0.62 |
|  |  |  | | Continent | |  | | |  |  | 0.84 | 0.22 | 0.24 |
|  |  |  | | Migratory strategy | |  | | |  |  | 0.08 | 0.01 | 0.81 |

^1)^ Migration route calculated in two steps
