# Supplementary material for: A New View on an Old Debate: Type of Cue-Conflict Manipulation and Availability of Stars Can Explain the Discrepancies between Cue-Calibration Experiments with Migratory Songbirds
Source: Front Behav Neurosci. 2016 Feb 23;10:29. doi: 10.3389/fnbeh.2016.00029 (PMC4763052; doi:10.3389/fnbeh.2016.00029)
Supplement: Supplementary file 3 [file Table3.DOCX]

**Supplementary table**

# Table S3. Cue calibration studies providing a full view of the surroundings during the cue conflict

| *Reference* | *Species* | *Continent* | *Migration strategy (distance)* | *Age* | *Season* | *Year* | *Latitude (°)* | *Longitude (°)* | *Shifted cue^1)^* | *Shift (°)* | *Total duration of cue conflict* |
| --- | --- | --- | --- | --- | --- | --- | --- | --- | --- | --- | --- |
| Bingman 1983 | Savannah sparrow | North America | short/medium | juv | autumn | 1980 | 46.6 | -73.8 | MF | -90 | ca. 2 months |
| Bingman 1984 | Pied Flycatcher | Eurasia | long | juv | autumn | 1982 | 50.1 | 8.7 | MF | 105 | ca. 2 months |
| Able & Able 1990 | Savannah sparrow | North America | short/medium | juv | autumn | 1988 | 46.6 | -73.8 | MF | 110 | 182 h |
| Able & Able 1990 | Savannah Sparrow | North America | short/medium | juv | autumn | 1988 | 46.6 | -73.8 | MF | 110 | 175 h |
| Able & Able 1990 | Savannah Sparrow | North America | short/medium | juv | autumn | 1988 | 46.6 | -73.8 | MF | 110 | 108 h |
| Prinz & Wiltschko 1992 | Pied flycatcher | Eurasia | long | juv | autumn | 1988 | 50.1 | 8.7 | MF | -120 | ca. 7 weeks |
| Able & Able 1995 | Savannah sparrow | North America | short/medium | juv+ad | autumn | 1994 | 46.6 | -73.8 | MF | (+ or -) 90 | 96 h |
| Able & Able 1995 | Savannah sparrow | North America | short/medium | juv+ad | autumn | 1994 | 46.6 | -73.8 | MF | (+ or -) 90 | 96 h |
| Able & Able 1997 | Savannah Sparrow | North America | short/medium | juv | autumn | 1995 | 46.6 | -73.8 | MF | 90 | >5 clear days |
| Weindler & Liepa 1999 | Pied flycatcher | Eurasia | long | ad | autumn | 1993 | 56.9 | 23.1 | MF | -90 | ca. 4 weeks |
| Åkesson et al. 2002 | White-crowned Sparrow | North America | long | juv | autumn | 1999 | 68.4 | -133.5 | MF | -90 | 60 min |
| Cochran et al. 2004 | Gray-cheeked thrush (*Catharus minimus*) | North America | long | ? | spring | 2003 | 40.1 | -88.2 | MF | 77 | >>60 min |
| Cochran et al. 2004 | Swaison’s thrush  (*Catharus ustulatus*) | North America | long | ? | spring | 2003 | 40.1 | -88.2 | MF | 86 | >>60 min |
| Muheim et al. 2006 | Savannah sparrow | North America | long | juv+ad | autumn | 2005 | 60.8 | -161.8 | PL | 90 ax | 60 min |
| Muheim et al. 2006 | Savannah sparrow | North America | long | juv+ad | autumn | 2005 | 60.8 | -161.8 | PL | 90 ax | 60 min |
| Wiltschko et al. 2008 | Australian silvereye (*Zosterops lateralis*) | Australia | short/medium | juv+ad | spring | 2007 | -30.5 | 151.6 | MF | -90 | 90+90 min |
| Muheim et al. 2009 | White-throated sparrow | North America | short/medium | juv+ad | spring | 2007 | 43.5 | -77.6 | PL | 90 ax | 60 min |
| Muheim et al. 2009 | White-throated sparrow | North America | short/medium | juv+ad | autumn | 2006 | 43.5 | -77.6 | PL | 90 ax | 60 min |
| Muheim et al. 2009 | White-throated sparrow | North America | short/medium | juv+ad | autumn | 2006 | 43.5 | -77.6 | MF | 90 | 60 min |
| Gaggini et al. 2010 | Pied flycatcher | Eurasia | long | ? | spring | 2007 | 40.8 | 13.4 | PL | 45 ax | 40 min |
| Gaggini et al. 2010 | Pied flycatcher | Eurasia | long | ? | spring | 2007 | 40.8 | 13.4 | MF | 90 | 40 min |
| Chernetsov et al. 2011 | Song thrush | Eurasia | short/medium | juv | autumn | 2010 | 55.1 | 20.8 | MF | 120 | >>60 min |
| Chernetsov et al. 2011 | Song thrush | Eurasia | short/medium | 2k^2)^ | spring | 2010 | 55.1 | 20.8 | MF | -120 | >>60 min |
| Schmaljohann et al. 2013 | Northern wheatear | Eurasia | long | juv | autumn | 2008 | 54.1 | 7.9 | PL | 90 ax | >60 min |
| Giunchi et al. 2014 | Pied flycatcher | Eurasia | long | ? | spring | 2011 | 40.8 | 13.4 | PL | 90 ax | 40 min |
| Giunchi et al. 2014 | Pied flycatcher | Eurasia | long | ? | spring | 2011 | 40.8 | 13.4 | PL | 90 ax | 40 min |
| Åkesson et al. 2015 | Dunnock | Eurasia | short/medium | juv | autumn | 2008 | 55.6 | 13.4 | MF | 90 | 60+60 min |
| Åkesson et al. 2015 | Robin | Eurasia | short/medium | juv | autumn | 2008 | 55.6 | 13.4 | MF | 90 | 60+60 min |
| Åkesson et al. 2015 | Robin | Eurasia | short/medium | juv | autumn | 2008 | 55.6 | 13.4 | MF | -90 | 60+60 min |
| this study | Garden warbler | Eurasia | long | juv | autumn | 2010 | 55.4 | 12.8 | MF | 90 | 60 min |

^1)^ MF = magnetic field; PL = polarized light

^2)^ 2k = second calendar year

# Table S3. continued…

| *Reference* | *Orientation cues available during cue conflict* | | | | *Type of orientation experiment* | *Orientation cues available during experiment* | *Magnetic compass response (*°*)* | *Celestial compass response (°)* | *Magnetic + star compass response (°)* | *Magnetic compass calibrated* | *Transfer of magnetic to celestial compass information* | *Magnetic/star compass calibrated* | *Comments* |
| --- | --- | --- | --- | --- | --- | --- | --- | --- | --- | --- | --- | --- | --- |
|  | *Sunrise* | *Sun* | *Sunset* | *Stars* |  |  |  |  |  |  |  |  |  |
| Bingman 1983 | Y | Y | Y | Y | funnel | MF | 90 ax |  |  | Y |  |  |  |
| Bingman 1984 | Y | Y | Y | Y | funnel | stars (VMF)^3)^ |  | 49 |  |  | (Y/N)? |  | Inconclusive: non-significant shift of star compass |
| Able & Able 1990 | Y | Y | Y | N | funnel | MF | 10 ax |  |  | N |  |  |  |
| Able & Able 1990 | Y | Y | Y | Y | funnel | MF | 34 ax |  |  | (Y)? |  |  | Inconclusive: sign., but only partial, shift of magnetic compass |
| Able & Able 1990 | N | N | N | Y | funnel | MF | 10 ax |  |  | N |  |  | No view of sunrise or sunset (exposure to night sky only) |
| Prinz & Wiltschko 1992 | Y | Y | Y | Y | funnel | MF | 119 |  |  | Y |  |  |  |
| Able & Able 1995 | Y | Y | Y | Y | funnel | MF | 91 ax |  |  | Y |  |  |  |
| Able & Able 1995 | Y | Y | Y | Y | funnel | MF | 101 ax |  |  | Y |  |  |  |
| Able & Able 1997 | Y | Y | Y | N | funnel | sunset (VMF) |  | 20 |  |  | N |  | Indirect evidence for magnetic compass calibration |
| Weindler & Liepa 1999 | Y | Y | Y | Y | funnel | MF | 67 |  |  | Y |  |  |  |
| Åkesson et al. 2002 | N | Y | N | N | funnel | MF/sunset |  |  | -87^4)^ |  | Y |  | No view of sunset during exposure (sun only); exposure to cue conflict in funnel prior to experiment |
| Cochran et al. 2004 | N | Y | Y | Y | radio tracking | MF/stars |  |  | -65 |  |  | Y |  |
| Cochran et al. 2004 | N | Y | Y | Y | radio tracking | MF/stars |  |  | -79 |  |  | Y |  |
| Muheim et al. 2006 | N | Y | Y | N | funnel | MF | 94 ax |  |  | Y |  |  |  |
| Muheim et al. 2006 | Y | Y | N | N | funnel | MF | 85 ax |  |  | Y |  |  |  |
| Wiltschko et al. 2008 | Y | Y | Y | Y | funnel | MF | -10 |  |  | N |  |  | Kept in outdoor aviary prior to exposure |
| Muheim et al. 2009 | N | Y | Y | N | funnel | MF | 88 ax |  |  | Y |  |  |  |
| Muheim et al. 2009 | Y | Y | N | N | funnel | MF | 79 ax |  |  | Y |  |  |  |
| Muheim et al. 2009 | N | Y | Y | N | funnel | MF | 100 ax |  |  | Y |  |  |  |
| Gaggini et al. 2010 | Y | Y | Y | (Y) | funnel | MF | -35 |  |  | (Y)? |  |  | Conflict between information at sunrise and sunset |
| Gaggini et al. 2010 | Y | Y | Y | (Y) | funnel | MF | -16 |  |  | N |  |  | Conflict between information at sunrise and sunset |
| Chernetsov et al. 2011 | N | Y | Y | Y | radio tracking | MF/stars |  |  | 17 |  |  | N | Kept in outdoor aviary prior to exposure; topographic bias? |
| Chernetsov et al. 2011 | N | Y | Y | Y | radio tracking | MF/stars |  |  | -3 |  |  | N | Kept in outdoor aviary prior to exposure; topographic bias? |
| Schmaljohann et al. 2013 | N | Y | Y | Y | radio tracking | MF/stars |  |  | -14 |  |  | N |  |
| Giunchi et al. 2014 | N | Y | Y | N | funnel | MF | 106 ax |  |  | Y |  |  |  |
| Giunchi et al. 2014 | N | Y | Y | N | radio tracking | MF/stars |  |  | 22 |  |  | N |  |
| Åkesson et al. 2015 | (Y) | Y | (Y) | N | funnel | MF | -10 |  |  | N |  |  | Cue-conflict exposure pre-sunset/sunrise |
| Åkesson et al. 2015 | (Y) | Y | (Y) | N | funnel | sunset (VMF) |  | 5 |  |  | N |  | Cue-conflict exposure pre-sunset/sunrise |
| Åkesson et al. 2015 | (Y) | Y | (Y) | N | funnel | sunset (VMF) |  | 33 |  |  | N |  | Cue conflict exposure pre-sunset/sunrise |
| this study | N | Y | Y | N | radio tracking | MF/stars |  |  | 17 |  |  | N |  |

^3)^ VMF = vertical magnetic field

^4)^ Response to magnetic and sunset cues
