# Supplementary material for: A New View on an Old Debate: Type of Cue-Conflict Manipulation and Availability of Stars Can Explain the Discrepancies between Cue-Calibration Experiments with Migratory Songbirds
Source: Front Behav Neurosci. 2016 Feb 23;10:29. doi: 10.3389/fnbeh.2016.00029 (PMC4763052; doi:10.3389/fnbeh.2016.00029)
Supplement: Supplementary file 4 [file DataSheet1.DOCX]

**Supplementary references**

Able, K. P., and Able, M. A. (1990). Ontogeny of migratory orientation in the Savannah sparrow, *Passerculus sandwichensis*: calibration of the magnetic compass. *Anim. Behav.* 39, 905–913.

Able, K. P., and Able, M. A. (1995). Interactions in the flexible orientation system of a migratory bird. *Nature* 375, 230–232.

Able, K. P., and Able, M. A. (1996). Migratory orientation: Autumn calibration of magnetic orientation is not evident in spring. *Naturwissenschaften* 83, 517–518.

Able, K. P., and Able, M. A. (1997). Development of sunset orientation cues in a migratory bird: no calibration by the magnetic field. *Anim. Behav.* 53, 363–368.

Åkesson, S., Morin, J., Muheim, R., and Ottosson, U. (2002). Avian orientation: effects of cue-conflict experiments with young migratory songbirds in the high Arctic. *Anim. Behav.* 64, 469–475.

Åkesson, S., Odin, C., Hegedüs, R., Ilieva, M., Sjöholm, C., Farkas, A., et al. (2015). Testing avian compass calibration: comparative experiments with diurnal and nocturnal passerine migrants in South Sweden. *Biol. Open* 4, 35–47. doi:10.1242/bio.20149837.

Bingman, V. P. (1983). Magnetic field orientation of migratory Savannah sparrows with different first summer experience. *Behaviour* 87, 43–51.

Bingman, V. P. (1984). Night sky orientation of migratory pied flycatchers raised in different magnetic fields. *Behav. Ecol. Sociobiol.* 15, 77–80.

Chernetsov, N., Kishkinev, D., Kosarev, V., and Bolshakov, C. V. (2011). Not all songbirds calibrate their magnetic compass from twilight cues: a telemetry study. *J. Exp. Biol.* 214, 2540–2543. doi:10.1242/jeb.057729.

Cochran, W. W., Mouritsen, H., and Wikelski, M. (2004). Migrating songbirds recalibrate their magnetic compass daily from twilight cues. *Science* 304, 405–408.

Gaggini, V., Baldaccini, N., Spina, F., and Giunchi, D. (2010). Orientation of the pied flycatcher, *Ficedula hypoleuca*: cue-conflict experiments during spring migration. *Behav. Ecol. Sociobiol.* 64, 1333–1342. doi:10.1007/s00265-010-0948-6.

Giunchi, D., Vanni, L., Baldaccini, N. E., Spina, F., and Biondi, F. (2014). New cue-conflict experiments suggest a leading role of visual cues in the migratory orientation of pied flycatchers, *Ficedula hypoleuca*. *J. Ornithol.* 156, 113–121. doi:10.1007/s10336-014-1107-z.

Muheim, R., Phillips, J. B., and Åkesson, S. (2006). Polarized light cues underlie compass calibration in migratory songbirds. *Science* 313, 837–839.

Muheim, R., Phillips, J. B., and Deutschlander, M. E. (2009). White-throated sparrows calibrate their magnetic compass by polarized light cues during both autumn and spring migration. *J. Exp. Biol.* 212, 3466–3472. doi:10.1242/jeb.032771.

Prinz, K., and Wiltschko, W. (1992). Migratory orientation of pied flycatchers: interaction of stellar and magnetic information during ontogeny. *Anim. Behav.* 44, 539–545.

Schmaljohann, H., Rautenberg, T., Muheim, R., Naef-Daenzer, B., and Bairlein, F. (2013). Response of a free-flying songbird to an experimental shift of the light polarization pattern around sunset. *J. Exp. Biol.* 216, 1381–1387. doi:10.1242/jeb.080580.

Weindler, P., and Liepa, V. (1999). The influence of premigratory experience on the migratory orientation of birds. in *Proc. 22nd Int. Ornithol. Congr.* (Johannesburg: Birdlife South Africa), 979–987.

Wiltschko, R., Munro, U., Ford, H., and Wiltschko, W. (2008). Contradictory results on the role of polarized light in compass calibration in migratory songbirds. *J. Ornithol.* 149, 607–614. doi:10.1007/s10336-008-0324-8.
